# Elevated levels of inflammatory plasma biomarkers are associated with risk of HIV infection

**DOI:** 10.1186/s12977-021-00552-6

**Published:** 2021-03-17

**Authors:** Samantha McInally, Kristin Wall, Tianwei Yu, Rabindra Tirouvanziam, William Kilembe, Jill Gilmour, Susan A. Allen, Eric Hunter

**Affiliations:** 1 Emory Vaccine Center at Yerkes National Primate Research Center, Atlanta, GA USA; 2 Rollins School of Public Health, Emory University, Atlanta, GA USA; 3 School of Data Science, The Chinese University of Hong Kong, Shenzhen, Shenzhen, Guangdong Province China; 4 Department of Pediatrics, Emory University School of Medicine, Atlanta, GA USA; 5 Center of CF and Airways Disease Research, Children’s Healthcare of Atlanta, Atlanta, GA USA; 6 Zambia-Emory HIV Research Project, Lusaka, Zambia; 7 Faculty of Medicine, Imperial College, London, SW7 2AZ UK; 8 Department of Pathology and Laboratory Medicine, Emory University, Atlanta, GA USA

**Keywords:** Cytokines, Chemokines, HIV pathogenesis, HIV acquisition, HIV discordant couples

## Abstract

**Background:**

To determine if individuals, from HIV-1 serodiscordant couple cohorts from Rwanda and Zambia, who become HIV-positive have a distinct inflammatory biomarker profile compared to individuals who remain HIV-negative, we compared levels of biomarkers in plasma of HIV-negative individuals who either seroconverted (pre-infection) and became HIV-positive or remained HIV-negative (uninfected).

**Results:**

We observed that individuals in the combined cohort, as well as those in the individual country cohorts, who later became HIV-1 infected had significantly higher baseline levels of multiple inflammatory cytokines/chemokines compared to individuals who remained HIV-negative. Genital inflammation/ulceration or schistosome infections were not associated with this elevated profile. Defined levels of ITAC and IL-7 were significant predictors of later HIV acquisition in ROC predictive analyses, whereas the classical Th1 and Th2 inflammatory cytokines such as IL-12 and interferon-γ or IL-4, IL-5 and Il-13 were not.

**Conclusions:**

Overall, the data show a significant association between increased plasma biomarkers linked to inflammation and immune activation and HIV acquisition and suggests that pre-existing conditions that increase systemic biomarkers represent a factor for increased risk of HIV infection.

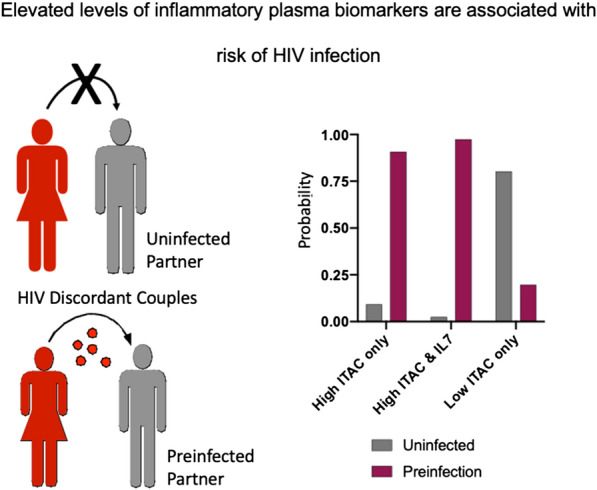

**Supplementary Information:**

The online version contains supplementary material available at 10.1186/s12977-021-00552-6.

## Background

HIV-1 remains a major health crisis facing the world today. At the end of 2019, 37.9 million people were infected with HIV-1 globally [1]. It is well known that sex workers, men who have sex with men, intravenous drug users, and transgender individuals are at increased risk for HIV acquisition [1], as are the seronegative partners in HIV-1 discordant cohabiting couples [2]. Genital inflammation and ulceration clearly contributes to increased risk of HIV-1 acquisition. Studies in discordant couples in Rwanda and Zambia, as well as studies in other cohorts, have shown that individuals with genital ulceration/inflammation have a 5-10-fold higher risk for HIV infection [3–10]. Genital ulceration/inflammation has also been shown to affect the transmission bottleneck in heterosexual HIV-1 infection. The presence of genital inflammation has been shown to increase the likelihood of transmission of two or more transmitted-founder viruses, or multivariate transmission [3, 11]. Sexually transmitted infections (STI) are frequently associated with increased genital inflammation. *Schistosoma haematobium*, which colonizes the venous plexus in the bladder, has also been shown to induce a similar increase in the risk of HIV acquisition [12–17]. In a study of the prevalence of *Schistosomiasis* antibodies in sera from a heterosexual HIV-discordant couple cohort in Lusaka, Zambia, it was found that Schistosome infections were linked to increased HIV-1 transmission in both sexes, increased acquisition of HIV-1 in women, and increased progression to death in HIV-positive women [18].

In a large study of at-risk HIV-negative women in South Africa it was observed that higher levels of inflammatory cytokines in the female genital tract of individuals prior to infection was associated with increased HIV susceptibility [6], allowing a more defined approach to quantitating this risk factor for acquisition. It is clear that in women the composition of the genital microbiome can also greatly influence the genital tract inflammatory state. Consistent with this notion, a study of the FRESH cohort in Durban, South Africa, showed that women who presented with *Lactobacillus*-deficient microbiota in their reproductive tract produced higher levels of inflammatory cytokines [19]. A follow-up study showed that these women had a greater risk of HIV acquisition compared to women with *Lactobacillus crispatus-rich* genital microbiota [20]. However, the genital cytokine levels observed were not correlated with those in the plasma [20].

In this study, we examined systemic plasma biomarker levels in individuals who would eventually seroconvert (pre-infection) and those who remained HIV-negative (uninfected) in a cohort of serodiscordant couples from both Zambia and Rwanda. We compared these two groups to determine if there was any association between systemic plasma biomarker levels and increased acquisition of HIV in HIV-negative individuals, and whether those biomarkers were associated with any pre-existing urogenital infections.

## Results

### Elevated systemic plasma cytokine and chemokine levels characterize individuals prior to infection

**Fig. 1:**
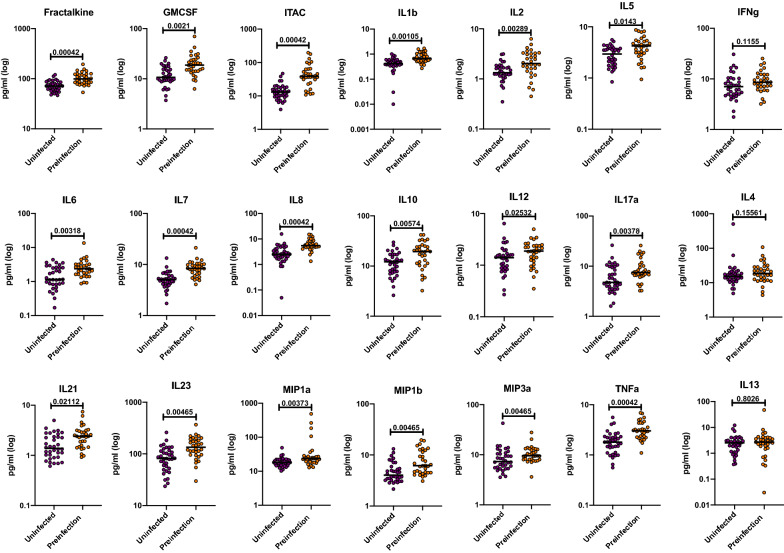
Circulating biomarkers in the combined Rwanda and Zambia cohorts: preinfection individuals have significantly higher cytokines and chemokine levels compared to the uninfected group. Cytokine and chemokines concentrations from plasma from the preinfection and uninfected groups were compared (Kolmogorov-Smirnov test, two-tailed, FDR adjusted p-values). The uninfected (purple) and preinfection (orange) groups contained a mixture of samples from Zambia and Rwanda

**Fig. 2:**
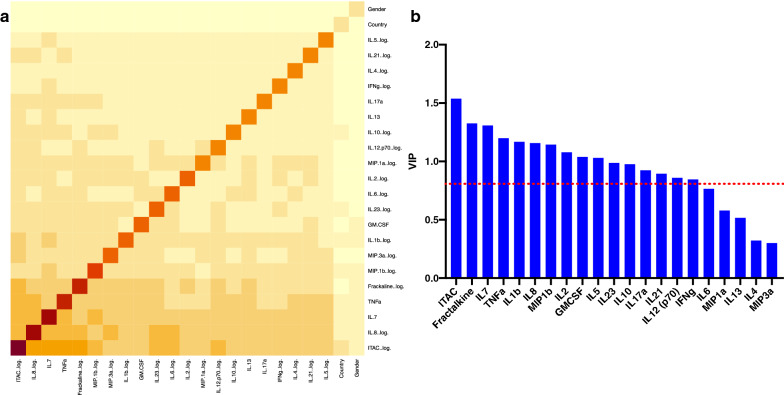
Partial least square (PLS) and Random Forest Analysis supported initial analyses. **a** Partial Least Square (PLS) Analysis for combined Zambian and Rwandan cohorts. Analysis was done with NIPALS Fit with 1 Factor. VIP (Variable Importance Plot) Threshold was set at 0.8. ITAC, GMCSF, Fractalkine, IFNg, IL-10, IL-12, IL-17a, IL-1b, IL-2, IL-21, IL-23, IL-5, IL-7, IL-8, MIP-1b, and TNFa were the biomarkers that caused preinfection profile in combined cohorts. **b** Random Forest Analysis used to determine which cytokines and chemokines contributed to the pattern of predisposition observed. In this analysis, the color of the square indicates the importance of the cytokine or chemokine to the profile of predisposition (light yellow is less importance, dark red is high importance)

A major goal of this study was to determine whether prior to infection seroconverting partners in the two discordant couple cohorts under study exhibited a different inflammatory cytokine or chemokine profile compared to those who remained seronegative. Plasma from a total of 38 Zambian participants (19 uninfected, 19 pre-infection) and 30 Rwandan participants (17 uninfected, 13 pre-infection) were analyzed in a Luminex multiplex assay. All of the individuals included in this study were negative partners in an HIV-1 serodiscordant couple, and were analyzed a median of > 1000 days following enrolment (Zambia) and > 450 days (Rwanda) (see Additional file 1: Table S1). Initial analyzes showed that the levels of the biomarkers were similar in the two countries, allowing an initial analysis of the combined Zambia and Rwanda cohorts (see Additional file 2: Figure S1). We observed that 18 of the 21 biomarkers measured were significantly increased in the pre-infection group compared to the uninfected group (Fractalkine, GMCSF, ITAC, IL-1ß, IL-2, IL-5, IL-6, IL-7, IL-8, IL-10, IL-12, IL-17a, IL-21, IL-23, MIP-1α, MIP-1ß, MIP-3α, and TNFα) (Fig. 1 and Additional file 3: Table S2). All p-values have undergone FDR-correction to account for the multiplicity of the assay. A majority of these upregulated cytokines and chemokines are involved in the inflammatory response. Because many of these cytokines are upregulated in coordinated pathways, we analyzed the data using a random forest model to establish those exerting most influence on the phenotype. Five biomarkers (ITAC, IL-8, IL-7, TNFα, and Fractalkine) were identified as the most significant contributors to the signature associated with future HIV-1 infection (Fig. 2a). We also analyzed the data using a Partial Least Squares (PLS) analysis. The PLS showed that the levels of GMCSF, Fractalkine, IFNg, ITAC, IL-1ß, IL-2, IL-5, IL-7, IL-8, IL-10, IL-12, IL-17α, IL-21, IL-23, MIP-1ß, and TNFα had higher impact on the separation between the uninfected and the pre-infection cohort (Fig. 2b and Additional file 4: Table S3 A). This result confirms our initial analysis that multiple inflammatory biomarkers are significantly elevated in individuals who later become infected. To further interrogate these findings, we analyzed the data from the Zambia and Rwanda combined cohorts to try and determine if genital inflammation contributed to the elevated profile observed in the pre-infection cohort.

### Presence of genital inflammation or ulceration in the combined cohort does not explain elevated biomarker levels in pre-infection group

**Fig. 3:**
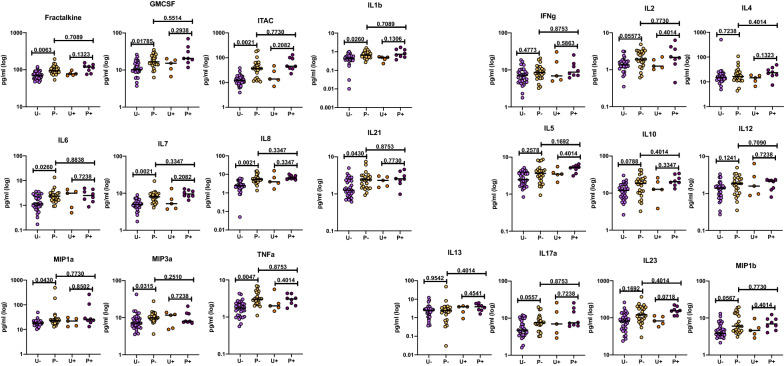
Preinfection individuals have significantly higher cytokines and chemokine levels compared to the uninfected group when individuals exhibiting genital inflammation/ulceration are excluded. Cytokine and chemokines concentrations from plasma from the Zambian and Rwandan preinfection and uninfected groups were compared when individuals were separated based on presence or absence of genital inflammation or ulceration. Forms of genital inflammation included: ulcers, inflammation due to STI, and inflammation not caused by STIs. The levels of cytokines and chemokines were separated by the country of origin (Kolmogorov-Smirnov test, two-tailed, FDR adjusted p-values). Light purple (uninfected individuals without genital inflammation, U−), yellow (preinfection individuals without genital inflammation, P−), orange (uninfected individuals with genital inflammation, U+), purple (preinfection individuals with genital inflammation, P+)

Past studies of the Zambian and Rwandan cohorts, as well as other studies, have shown that the presence of genital inflammation/ulceration increases an individual’s susceptibility to infection by HIV [3, 4, 6–9, 19, 20]. To test the possible role of genital inflammation or ulceration in the elevated biomarker profile, we compared the levels in individuals from the pre-infection group for whom any form of genital inflammation or ulceration had been reported in the 6 months prior to sample collection with those for whom no genital inflammation or ulceration was reported. There was no significant difference in the levels of any of the 21 analytes analyzed. Similarly, genital inflammation/ulceration did not significantly impact systemic biomarkers in the uninfected group. Moreover, after removing individuals with genital inflammation from the analysis, 11 of the biomarkers remained significantly higher in the pre-infection group compared to the uninfected group (Fractalkine, GMCSF, ITAC, IL-1ß, IL-6, IL-7, IL-8, IL-21, MIP-1α, MIP-3α, and TNFα) (Fig. 3). We next analyzed the Rwandan and Zambian cohorts separately to determine whether any differences existed between the two cohorts when analyzed in a similar manner.

### Elevated systemic plasma cytokine and chemokine levels associated with pre-infection individuals in both Zambian and Rwandan cohorts

**Fig. 4:**
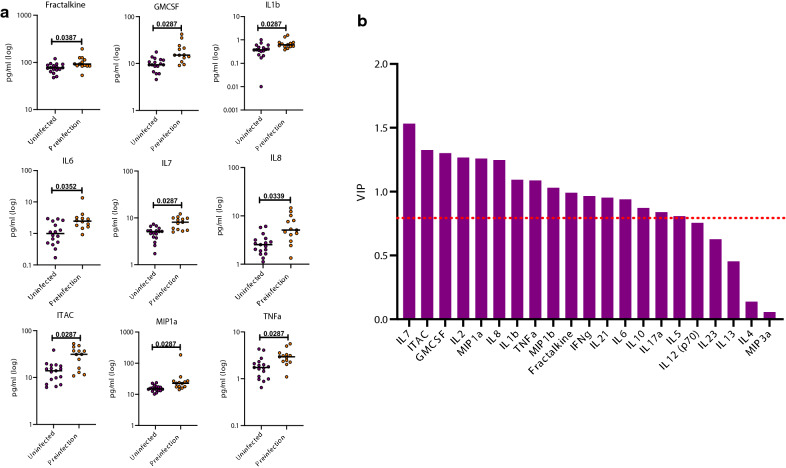
Circulating biomarkers in the Rwanda cohort: preinfection individuals have significantly higher cytokines and chemokine levels compared to the uninfected group. **a** The levels of cytokines and chemokines were separated by the country of origin and the uninfected (purple) and preinfection (orange) groups were compared (Kolmogorov-Smirnov test, two-tailed, FDR adjusted p-values). **b** Partial Least Square (PLS) Analysis for the Rwandan cohort. Analysis was done with NIPALS Fit with 1 Factor. VIP (Variable Importance Plot) Threshold was set at 0.8. PLS analysis for Rwandan cohort. ITAC, GMCSF, Fractalkine, IFNg, IL-10, IL-17a, IL-1b, IL-2, IL-21, IL-5, IL-6, IL-7, IL-8, MIP-1a, MIP-1b, and TNFa were the biomarkers that caused preinfection profile in Rwanda

Despite the smaller numbers analyzed when the data was separated by country, we still observed that several inflammatory biomarkers were significantly higher in the pre-infection group than the uninfected group. In Rwanda, Fractalkine, GMCSF, ITAC, IL-1ß, IL-6, IL-7, IL-8, MIP-1α, and TNFα concentrations were significantly increased in the pre-infection group compared to the uninfected group (Fig. 4a and Additional file 5:  Table S4A). The median, interquartile range, and p-values for this analysis is shown in Additional file 5: Table S4A. When analyzed in a PLS model, Fractalkine, GMCSF, IFNg, ITAC, IL-1ß, IL-2, IL-5, IL-6, IL-7, IL-8, IL-10, IL-17α, IL-21, MIP-1α, MIP-1ß, and TNFα were found to be the major contributors to the separation of the groups (Fig. 4b and Additional file 4: Table S3B). This supports our initial univariate analysis of the Rwanda cohort since there were a number of additional biomarkers that were trending towards significance (see Additional file 6:  Figure S2).

**Fig. 5:**
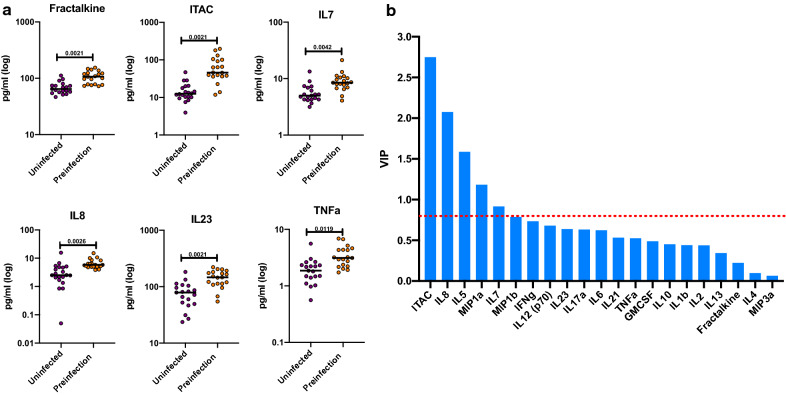
Circulating biomarkers in the Zambia cohort: preinfection individuals have significantly higher cytokines and chemokine levels compared to the uninfected group. **a** The levels of cytokines and chemokines were separated by the country of origin and the uninfected (purple) and preinfection (orange) groups were compared (Kolmogorov-Smirnov test, two-tailed, FDR adjusted p-values). **b** Partial Least Square (PLS) Analysis for the Zambian cohort. Analysis was done with NIPALS Fit with 1 Factor. VIP (Variable Importance Plot) Threshold was set at 0.8. **a** PLS analysis for Zambian cohort. ITAC, IL-5, IL-8, and MIP-1a were the biomarkers that caused preinfection profile in Zambia

In Zambia, Fractalkine, ITAC, IL-7, IL-8, IL-23, and TNFα concentrations were significantly increased in the pre-infection group compared to the uninfected group in univariate comparisons (Fig. 5a and Additional file 7:  Figure S3). The median, interquartile range, and p-values for this analysis are shown in Additional file 5: Table S4B. While elevated levels of Fractalkine, ITAC, IL-7, IL-8, and TNFα are observed for both countries in their respective pre-infection groups, they differ in that they exhibit additional biomarkers unique to each country. The PLS analysis showed that levels of ITAC, IL-5, IL-7, IL-8, and MIP-1α were associated with pre-infection (Fig. 5b and Additional file 4: Table S3C).

Taken together, we observed that the pre-infection group had elevated biomarker levels in both Rwanda and Zambia even when the cohorts are analyzed separately.

### Presence of *Schistosomiasis *antibody titers does not appear to be a major contributor to the elevated biomarker profile in pre-infected individuals

We have recently reported that infection with *Schistosoma haematobium* is common even in urban settings in Zambia and that infection was associated with increased susceptibility to HIV-1 infection [18]. Because this parasite colonizes the venous plexus in the bladder, we investigated prior infection as evidenced by antibodies resulted in elevated cytokines. However, screening of the plasma from both the uninfected and pre-infection groups for antibodies to the schistosome showed that a similar proportion of individuals in both had detectable antibody titers (9/19 uninfected; 11/19 pre-infection). The Zambian cohort was therefore divided into four groups: uninfected individuals with negative *Schistosomiasis* antibody titers, uninfected individuals with positive antibody titers, pre-infection individuals with negative titers, and pre-infection individuals with positive titers (see Additional file 8: Figure S4). We did not observe any significant differences in biomarker levels between seropositive and seronegative individuals in either the uninfected group or the pre-infection group. With the proviso that we are analyzing very small groups of individuals, this suggests that schistosome infection is not associated with the elevated biomarker levels observed in the pre-infection group.

### Predictive analyses identify elevated levels of several biomarkers as markers of pre-infection

**Fig. 6:**
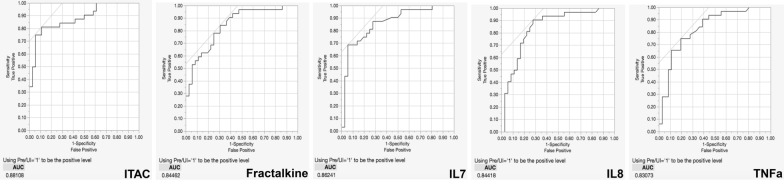
Receiver Operating Characteristics (ROC) curves for combined Zambia and Rwanda cohorts identifies biomarkers that distinguishes preinfection individuals. Elevated levels of ITAC, Fractalkine, IL-7, IL-8, and TNFa identify individuals as risk for HIV acquisition. Area under the curve (AUC) shut off was 0.8 for separating the uninfected and preinfection individuals

**Fig. 7:**
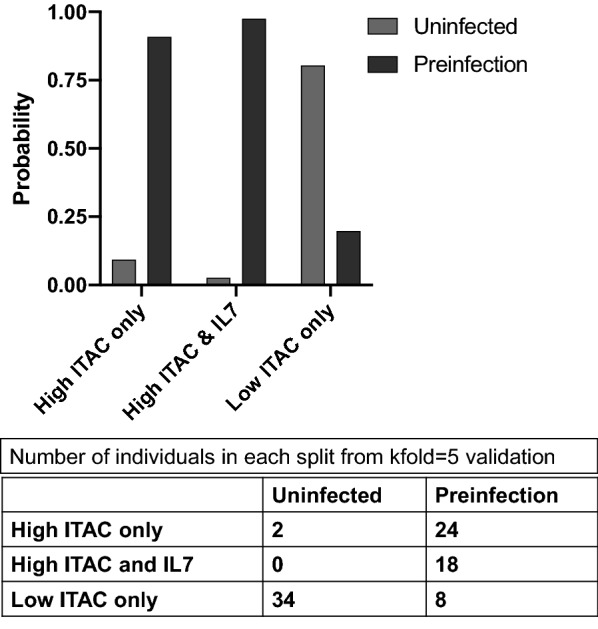
Kfold validation model identifies elevated ITAC and IL-7 levels as markers for preinfection from combined Rwandan and Zambian cohorts. Two splits were performed to find biomarkers associated with preinfection. The bar graph shows the response probability for the multiple splits performed in the model. The model found that elevated ITAC identified preinfection individuals with a probability of 0.9077 and elevated ITAC and IL-7 identified preinfection individuals with a probability of 0.9744. Lower levels of ITAC were found identify uninfected individuals. Uninfected (gray) and preinfection (maroon). The table shows the number of individuals from the total cohort that fall into each split performed by the model (kfold = 5)

Based on preliminary results from partial least squares analysis (Figs. 2b, 4b and 5b), we wanted to determine which biomarkers included in the study were associated with future HIV-1 acquisition. To achieve this, we analyzed the combined Zambian and Rwandan cohort using Receiver Operating Characteristics (ROC) curves and established a cutoff for the area under the curve (AUC) of 0.8. Using a cutoff of 0.8 means that any biomarker identified in the analysis has over an 80 % chance of distinguishing pre-infection individuals from those that remain uninfected. ITAC, Fractalkine, IL-7, IL-8, and TNFα were identified as markers predictive for pre-infection (Fig. 6). ROC curves for individual Zambia and Rwanda cohorts are shown in Additional files 9 and 10: Figures S5 and S6. This finding is supported by our random forest analysis (Fig. 2a). Using a kfold validation model, we found that concentrations of ITAC above 30.98 pg/ml was highly predictive of a seronegative partner who would become infected (probability of 0.91; sensitivity of 0.75; specificity 0.94). If an IL-7 value above 6.99 pg/ml was added to the model, the probability of identifying pre-infection individuals increased to 0.97 (Fig. 7).

## Discussion

This study found that individuals who will eventually seroconvert have higher levels of proinflammatory biomarkers in their plasma compared to individuals who remain HIV-negative, suggesting a novel link between predisposition to HIV infection and systemic biomarker levels.

Our findings suggest that pre-existing conditions which induce systemic inflammation can represent a risk factor for HIV acquisition.

An example of a viral infection capable of inducing this is hepatitis C virus. Plasma levels of IL-10 and MIP1ß were positively correlated with HCV RNA levels and may be involved in HCV immunopathogenesis [21], HIV/HCV co-infected women had higher levels of several proinflammatory biomarkers – biomarkers elevated in each infection or disease discussed in this section are summarized in Additional file 11: Table S5. In addition, HCV-positive HIV-negative women had higher levels of IFNg and IL-17 compared to other groups [22]. However, the prevalence of HCV infections in both Zambia and Rwanda has been reported to be low [23, 24].

Elevated systemic levels of cytokines and chemokines are also seen in multiple infections common in sub-Saharan Africa. Multiple researchers have found that malaria infection has been linked to elevated systemic cytokine responses. HIV and *P. falciparum* co-infected individuals had increased HIV viral loads, a steeper decline in CD4^+^ T cell counts, and exhibited higher levels of several acute phase proinflammatory cytokines [25]. Similarly, symptomatic malaria infections had increased levels of multiple cytokines compared to uninfected controls [26]. It has also been found that individuals with greater malaria disease severity had higher levels of CRP, TNFα, and IFNg [27]. *P. vivax* infections also are associated with higher levels of proinflammatory cytokines [28].

Elevated systemic biomarkers have also been found in Tuberculosis (TB). Multiple studies found that individuals with acute and latent TB infections had higher levels of cytokines and chemokines compared to healthy controls [29–32]. These results show that TB infections can increase several proinflammatory cytokines regardless of the stage of infection.

A separate study looked at proinflammatory cytokine levels in TB-infected individuals co-infected with *S. stercoralis*. *S. stercoralis* is a soil-transmitted helminth that infects about 50–100 million people worldwide [33]. Individuals co-infected with TB and *S. stercoralis* have increased levels of several type 2 cytokines compared to individuals only infected with TB [34]. One possible explanation for this is an association with microbial translocation. *S. stecoralis* infected individuals exhibited significantly higher plasma levels of microbial translocation markers (i.e. LPS) and this was associated with increased levels of several proinflammatory cytokines [35]. Researchers looking at microbial translocation in HIV infected individuals found that LPS was also positively correlated with plasma levels of IL-6, TNFα, and hsCRP [36]. This finding is particularly interesting because it is well known that microbial translocation is associated with disease severity in HIV infections [37].

These studies show that several common infections in sub-Saharan Africa are capable of elevating systemic levels of several pro-inflammatory cytokines, which we found as a risk factor for HIV acquisition. However, it is also possible that higher levels of biomarkers prior to infection may have additional impact once an individual becomes HIV-infected. High levels of several cytokines appear to enhance HIV replication and disease progression [38]. Additional studies are needed not only to determine the cause of the elevated levels of biomarkers in individuals prior to HIV acquisition, but also how these elevated levels may impact the disease progression of these patients once infected.

While these common infections in Africa are associated with elevated inflammatory cytokines, the observed biomarker profile of the pre-infection individuals reported on here is not indicative of a classic innate immune response or T cell response. Indeed, the elevated cytokines and chemokines observed play roles in many different arms of the immune system. Our analysis showed that three biomarkers, Fractalkine, ITAC, and IL-7, were highly predictive as risk factors for HIV acquisition if elevated above certain levels. These biomarkers can be elevated from infection, but also from non-communicable conditions. Compared to healthy controls, elevated levels of Fractalkine were found in individuals with type 2 diabetes, systemic lupus erythematosus, and systemic sclerosis, an autoimmune disorder that affects the vascular system and leads to early defective angiogenesis. [39–42]. In systemic sclerosis, the higher levels of soluble Fractalkine in the blood were associated with vascular activation and increased disease severity [42]. That same study found higher levels of ITAC compared to healthy controls [42]. ITAC, also known as IFN-inducible T cell α chemoattractant, was found to be at higher levels in patients with inflammatory bowel disease (Crohn’s disease and ulcerative colitis), fibromyalgia, and sarcoidosis [43–45]. Sarcoidosis is a systemic inflammatory granulomatous disease that affects lungs of its patients and higher systemic levels of ITAC were found to be predictive of future pulmonary function test decline [43].

IL-7 is not normally considered an inflammatory cytokine and is more associated with T cell homeostasis. However, higher levels of IL-7 were found to be associated with several diseases, such was fibromyalgia [46] and also in patients with colorectal and esophageal cancers compared to healthy controls [47]. Unfortunately, health data on such conditions were not recorded as part of the Heterosexual Transmission Protocol into which discordant couples were enrolled. Nevertheless, the studies reported here indicate that additional studies of cytokine/chemokine levels in at risk individuals is warranted.

There are a number of limitations to the current study. We were only able to analyze the inflammatory biomarkers in a limited number of individuals since the availability of pre-HIV infection plasma samples from the discordant couples studied here are also limited (only 3–7 % of individuals in this cohort seroconverted per year depending on the cohort). In addition, we have limited information on other infections and health conditions of individuals in study. This is compounded by the fact that only a small number of studies have reported on systemic biomarkers in other infections or diseases in the context of an African population. As a result, it is difficult for us to identify specific infections or diseases that may be causing the elevated biomarker profile that we observed. Analogous studies of cohorts in the USA or Europe, where additional biomarker data on other infections/diseases may be available, would represent a valuable follow-up study to this one.

The identification of key biomarkers associated with HIV acquisition has important clinical ramifications. Our study identified elevated levels of both ITAC and IL-7 as highly predictive of HIV acquisition risk. If individuals in a HIV high-risk region are found to have elevated levels of one or both of these biomarkers, more intensive HIV-1 prevention approaches could be taken in order to protect those individuals. Additional studies are needed not only to determine the cause of the elevated levels of biomarkers in individuals prior to HIV acquisition and how they facilitate the acquisition event, but also how these elevated levels may impact the disease progression of these patients once infected. Overall, our findings suggest that individuals at risk for infection could be identified by testing for elevated levels of a very limited number of biomarkers.

## Conclusions

We show that in a cohort of seronegative partners from serodiscordant couples, individuals who eventually acquire HIV from their partner have higher levels of inflammatory cytokines and chemokines compared to individuals that remain HIV-negative. This was observed in both Rwanda and Zambia where subtype A and subtype C are the predominant HIV-1 serotypes. A Receiver Operator Characteristics analysis showed that the levels of just two cytokines, ITAC and IL-7, were highly predictive of future infection. This suggests high systemic biomarker levels are both a risk factor and a quantitative predictor for HIV acquisition.

## Materials and methods

### Study Subjects

All participants were enrolled in the Rwanda Zambia HIV Research Group (RZHRG) discordant couple cohorts in Lusaka, Zambia and Kigali, Rwanda. Subjects from both cohorts were enrolled in human subjects protocols approved by the Emory Institutional Review Board, the Rwanda National Ethics Committee and the University of Zambia Research Ethic Committee and provided written consent. When the participants enrolled in the cohort, they were provided couples counseling and testing, treatment for sexually transmitted infections (STIs), and condoms to reduce transmission of HIV-1. All the subjects tested in this study were the seronegative partner within a serodiscordant cohabitating heterosexual couple. Subjects were selected on their seronegative status, availability of plasma samples, and selected from both Zambian and Rwandan cohorts. The negative partner was tested for HIV every 1 to 3 months. In the Zambia cohort, the median days from enrollment to when the sample was taken was 1234 for the uninfected group and 1087 for the pre-infection group. Samples for the pre-infection group was collected a median of 46 days before the estimated date of infection (EDI). In the Rwanda cohort, the median days from enrollment to when the samples were taken was 494 for the uninfected group and 457 for the pre-infection group. Samples for the pre-infection group was collected a median of 45 days before the EDI. The algorithm used to determine the EDI has been previously described [3].

### Evaluation of plasma cytokines

The plasma cytokine and chemokine levels were measured using a Milliplex Map Human High Sensitivity T Cell Panel (HSTCMAG-28SK). This kit measures the levels of 21 inflammatory cytokines and chemokines. The samples were run in duplicate. In order to eliminate batch to batch variation in the assay, all tests were carried out on the same batch of plates and approximately equal numbers of pre-infection and uninfected plasma were run on the same plate. The plates were quantified and standardized on a Bioplex 2000 at the Yerkes Virology Core and final concentrations were extrapolated from a standard curve and expressed in pg/ml. All plasma samples were stored at -80 °C and had undergone a single freeze-thaw for aliquoting prior to use.

### Genital inflammation and ulceration data collection

As described in Haaland et al. [3], medical and laboratory signs and symptoms of inflammatory or ulcerative STI, candida, and bacterial vaginosis were recorded systematically at routine study visits and at interim sick visits, with full physical and/or genital exams conducted annually and as clinically indicated; physical and genital exams were routinely conducted on the visit date when lab test results indicated HIV-1 seroconversion. A self-reported symptom was considered present whether or not the patient sought medical treatment and included treatment administered at external clinics. The generation of the composite variables were described in Wall et al. [4]. Briefly, for each 3-monthly interval, composite variables were created. The genital inflammation composite included inflammatory STIs (clinical or laboratory diagnosis or treatment of gonorrhea, chlamydia or trichomonas) and non-inflammatory STIs (reported discharge, dysuria, dyspareunia; observed discharge or inflammation of external or internal genitalia; and/or laboratory diagnosis of candida or BV). The composite for genital ulcer included observed or reported genital ulcers and/or incident positive RPR. A subject was considered having positive genital inflammation or ulceration if they had presence of either in the 6 months prior to biomarker sample collection.

### *Schistosomiasis *antibody titer data collection

As described in Wall et al. [18], plasma samples were collected at baseline analyzed in an ELISA assay for antibodies to schistosome soluble worm antigen preparation (SWAP). A 4-parameter curve fitting model was used to assign units based on the standard curve to each unknown plasma. The positive cutoff value was set at three standard deviations above the average anti-SWAP IgG in serum from egg negative controls from the US and Europe. A positive schistosomiasis result was defined as having a positive SWAP antibody response.

### Data analysis

Comparison between the uninfected and the pre-infection groups were done with nonparametric Kolmogorov-Smirnov tests in Prism 9. We addressed the multiple testing issue by using the Benjamini-Hochberg False Discovery Rate (FDR) correction [48]. The Random Forest model was performed in R 4.0.0 at the default setting. The details were described previously [49].

Partial Least Squares (PLS) analysis was performed using the JMP Pro 15 statistical package. PLS analysis had a variable importance cutoff of 0.8. The Receiver Operating Characteristic (ROC) curve analysis used a cutoff of 0.8 for area under the curve (AUC). The predictive results were generated with a k-fold validation which included two splits in the model (k = 5).

##  Supplementary Information

**Additional file 1 Table S1. **Demographics of Zambia and Rwanda cohort.

**Additional file 2: Figure S1.** Uninfected and Preinfection levels of cytokines and chemokines compared between Zambia and Rwanda. There were no significant differences observed between levels of biomarkers in Zambia (blue) and Rwanda (purple) in both the uninfected and preinfection cohorts.

**Additional file 3: Table S2. **Biomarkers increased in preinfection group compared to uninfected group in combined Rwandan and Zambian cohort.

**Additional file 4: Table S3.** Variable Importance values for Partial Least Square analyses for combined cohorts (A), Rwandan cohort (B), and Zambian cohort (C).

**Additional file 5: Table S4.** Biomarkers increased in preinfection group compared to uninfected group in Rwandan cohort (A) and the Zambian cohort (B).

**Additional file 6: Figure S2. **Nonsignificant circulating biomarkers in the Rwanda cohort: preinfection individuals nonsignificant levels cytokines and chemokine levels compared to the uninfected group. The levels of cytokines and chemokines of the uninfected (purple) and preinfection (orange) groups were compared (Kolmogorov-Smirnov test, two-tailed, FDR adjusted p-values).

**Additional file 7: Figure S3.** Nonsignificant circulating biomarkers in the Zambia cohort: preinfection individuals have nonsignificant levels of cytokines and chemokine levels compared to the uninfected group. The levels of cytokines and chemokines were separated by the country of origin and the uninfected (purple) and preinfection (orange) groups were compared (Kolmogorov-Smirnov test, two-tailed, FDR adjusted p-values).

**Additional file 8: Figure S4.** *Schistosomiasis* infections in Zambian cohort does not appear to a major contributor to elevated biomarker profile. Zambian preinfection individuals with positive *Schistosomiasis* antibody titers do not have significantly higher cytokine levels than individuals with negative *Schistosomiasis* antibody titers regardless of uninfected or preinfection status. Levels of biomarkers were compared between three groups: uninfected individuals with negative antibody titers (U-, light blue), preinfection individuals with negative antibody titers (P-, pink), uninfected individuals with positive titers (U+, dark blue) and preinfection individuals with positive antibody titers (P+, magenta) (Kolmogorov-Smirnov test, two-tailed, unadjusted p-values).

**Additional file 9: Figure S5. **Receiver Operating Characteristics (ROC) curves for Zambia cohort identifies biomarkers that distinguishes preinfection individuals. Elevated levels of ITAC, Fractalkine, IL-23, IL-7, IL-8, and TNFa identify individuals as risk for HIV acquisition. Area under the curve (AUC) shut off was 0.8 for separating the uninfected and preinfection individuals.

**Additional file 10: Figure S6. **Receiver Operating Characteristics (ROC) curves for Rwanda cohort identifies biomarkers that distinguishes preinfection individuals. Elevated levels of ITAC, GMCSF, IL-1b, IL-6, IL-7, IL-8, MIP-1a, and TNFa identify individuals as risk for HIV acquisition. Area under the curve (AUC) shut off was 0.8 for separating the uninfected and preinfection individuals.

**Additional file 11: Table S5. **Elevated biomarkers and diseases/infections mentioned in Discussion.

## Data Availability

Source data available on request.
