# Supplementary material for: Elevated levels of inflammatory plasma biomarkers are associated with risk of HIV infection
Source: Retrovirology. 2021 Mar 17;18:8. doi: 10.1186/s12977-021-00552-6 (PMC7968240; doi:10.1186/s12977-021-00552-6)
Supplement: Supplementary file 1 — Additional file 1 Table S1. Demographics of Zambia and Rwanda cohort. [file 12977_2021_552_MOESM1_ESM.pdf]

	Zambia	Rwanda
Total # of individuals in cohort	38	30
# in the uninfected cohort	19	17
# of males	8	7
# of females	11	10
# with genital inflammation/ulceration	3	2
# with positive Schistosomiasis titers	1	3
Median days from enrollment	1234	494
# in the preinfection cohort	19	13
# of males	11	7
# of females	8	6
# with genital inflammation/ulceration	7	1
# with positive Schistosomiasis titers	11	2
Median days from enrollment	1087	457
Median days before infection	46	45
