# Supplementary figures and images for: Elevated levels of inflammatory plasma biomarkers are associated with risk of HIV infection
Source: Retrovirology. 2021 Mar 17;18:8. doi: 10.1186/s12977-021-00552-6 (PMC7968240; doi:10.1186/s12977-021-00552-6)

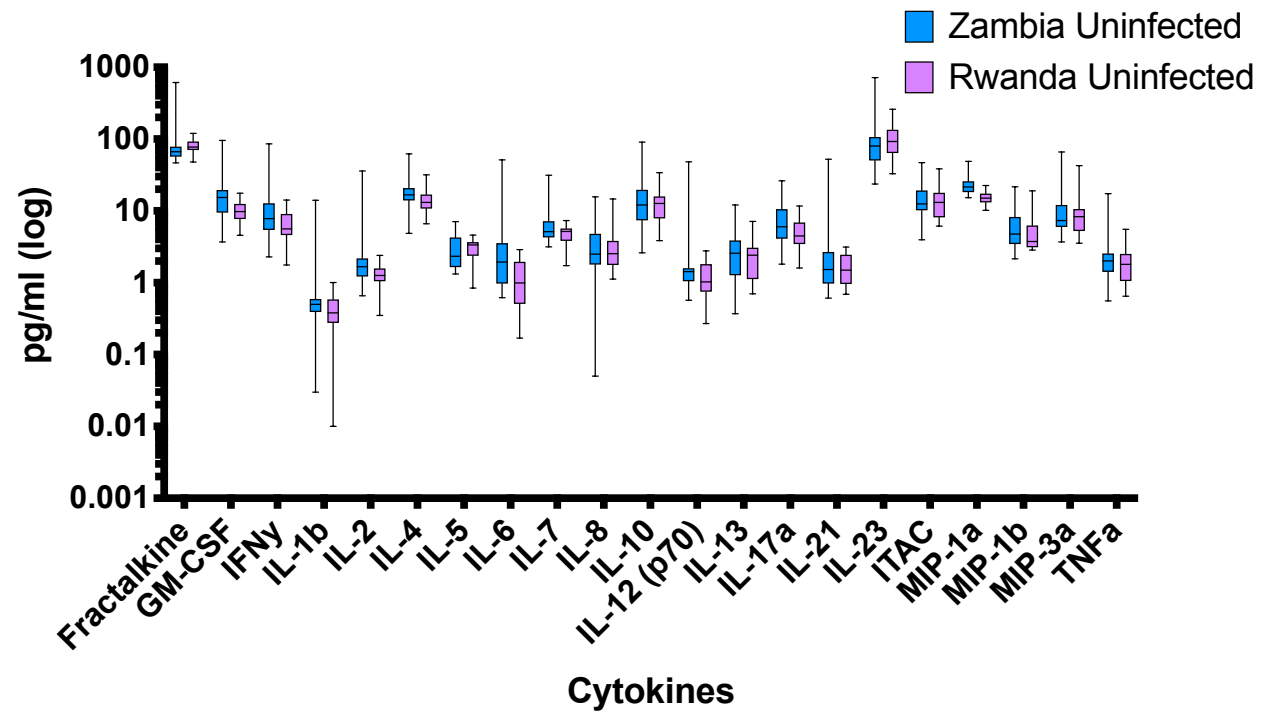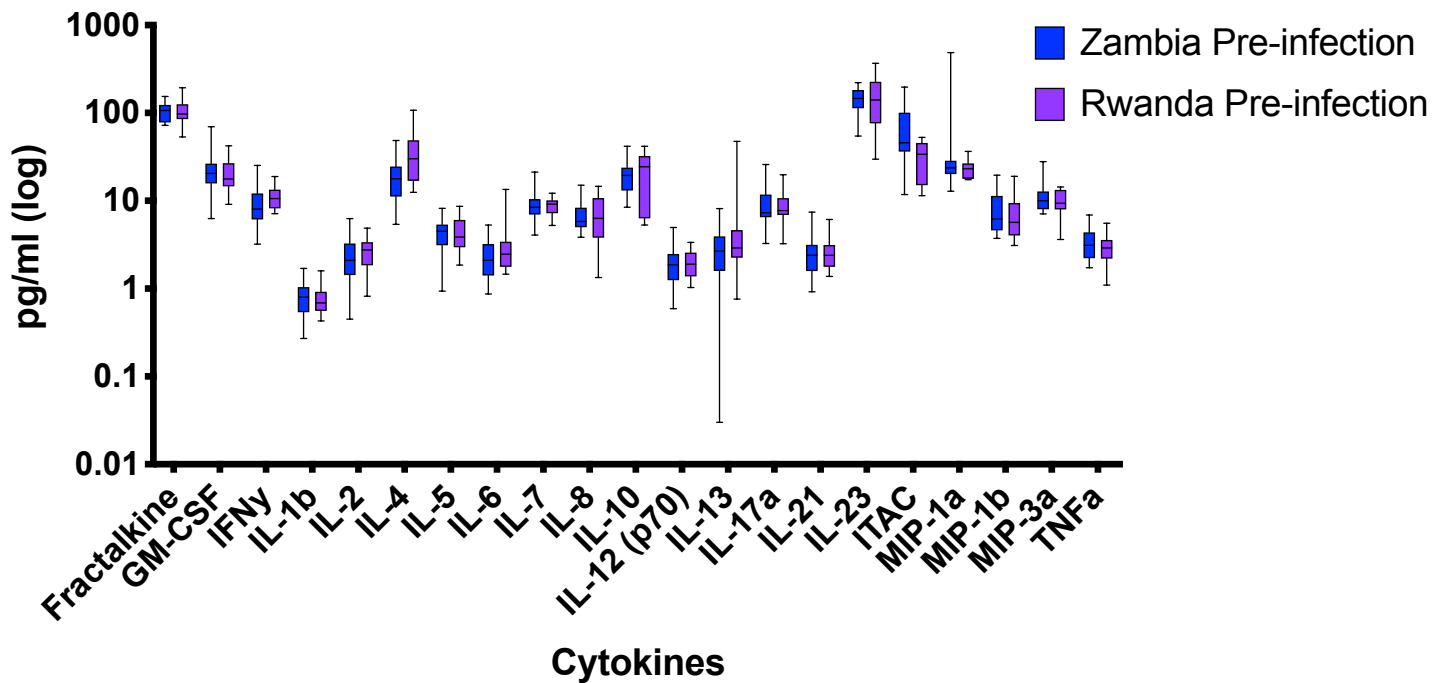

Supplement: Supplementary file 2 — Additional file 2: Figure S1. Uninfected and Preinfection levels of cytokines and chemokines compared between Zambia and Rwanda. There were no significant differences observed between levels of biomarkers in Zambia (blue) and Rwanda (purple) in both the uninfected and preinfection cohorts. [file 12977_2021_552_MOESM2_ESM.pdf]

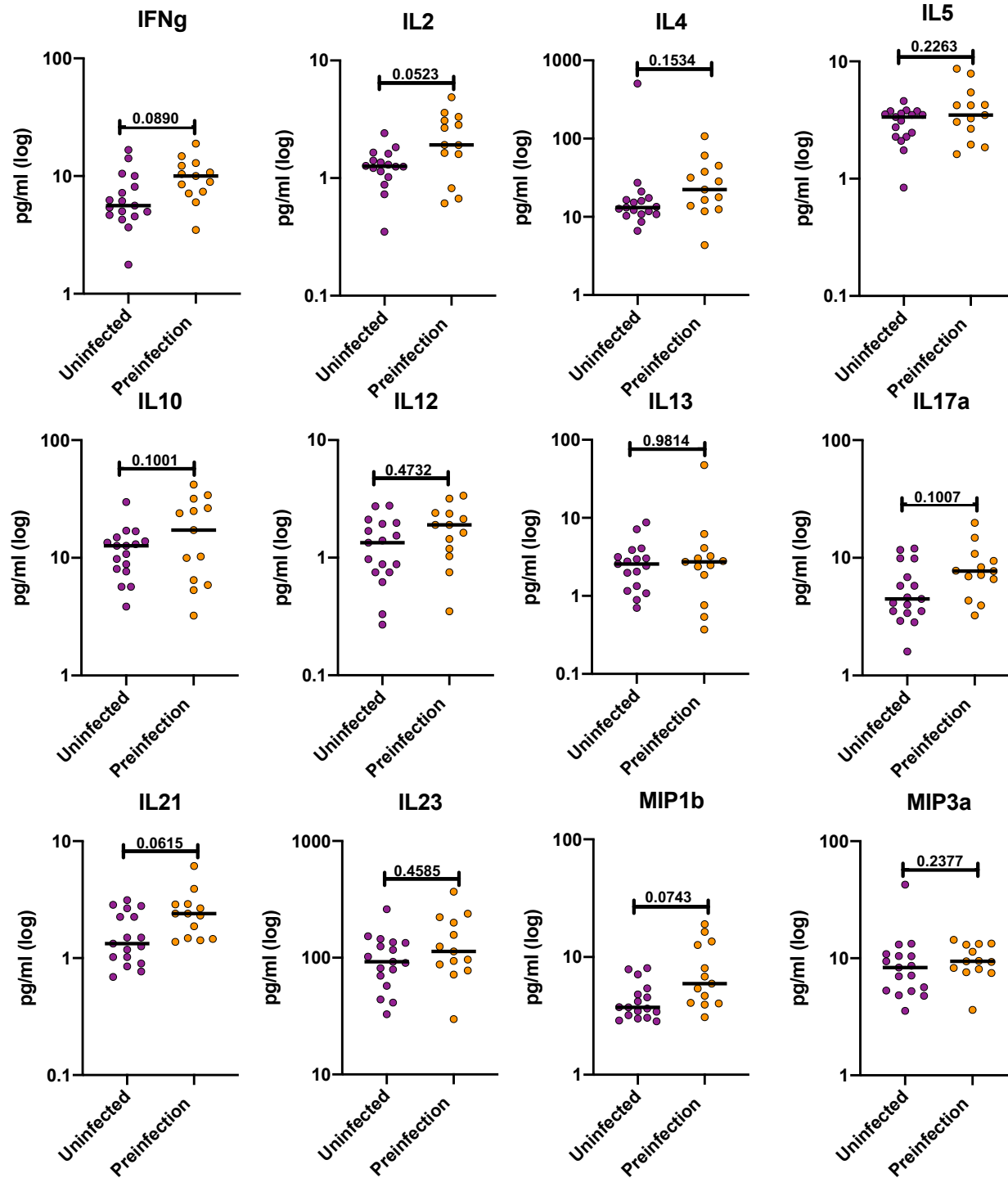

Supplement: Supplementary file 6 — Additional file 6: Figure S2. Nonsignificant circulating biomarkers in the Rwanda cohort: preinfection individuals nonsignificant levels cytokines and chemokine levels compared to the uninfected group. The levels of cytokines and chemokines of the uninfected (purple) and preinfection (orange) groups were compared (Kolmogorov-Smirnov test, two-tailed, FDR adjusted p-values). [file 12977_2021_552_MOESM6_ESM.pdf]

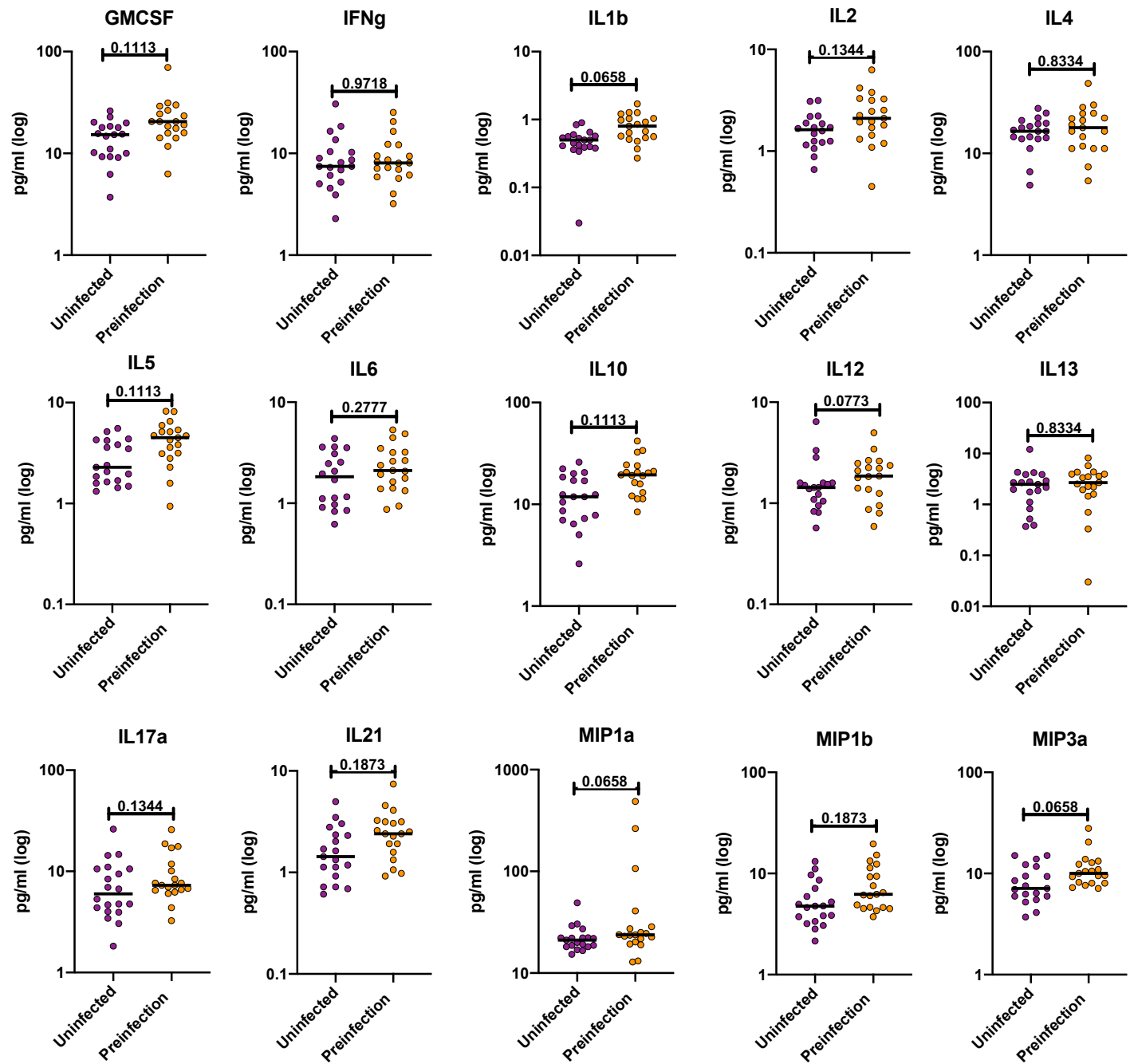

Supplement: Supplementary file 7 — Additional file 7: Figure S3. Nonsignificant circulating biomarkers in the Zambia cohort: preinfection individuals have nonsignificant levels of cytokines and chemokine levels compared to the uninfected group. The levels of cytokines and chemokines were separated by the country of origin and the uninfected (purple) and preinfection (orange) groups were compared (Kolmogorov-Smirnov test, two-tailed, FDR adjusted p-values). [file 12977_2021_552_MOESM7_ESM.pdf]

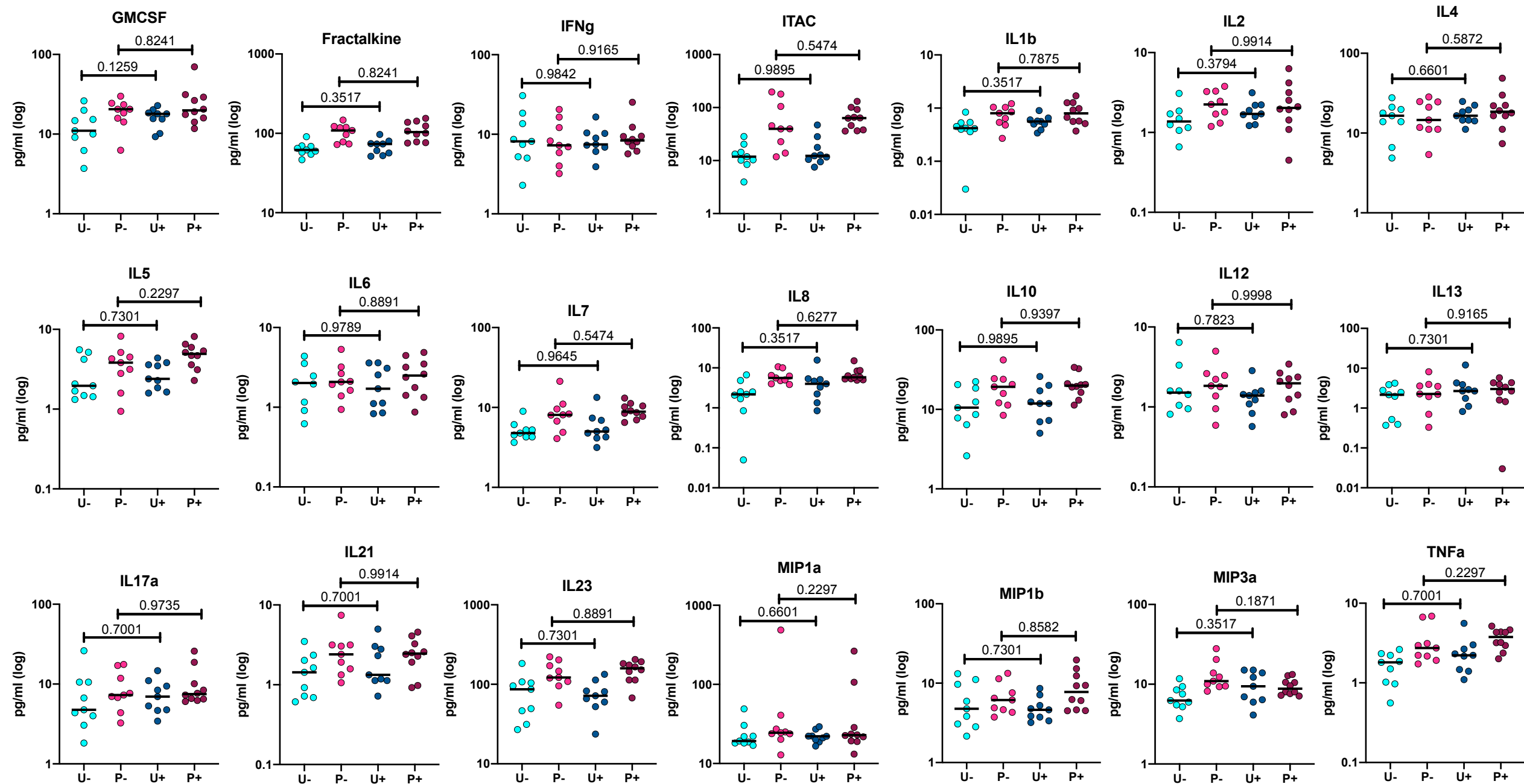

Supplement: Supplementary file 8 — Additional file 8: Figure S4. Schistosomiasis infections in Zambian cohort does not appear to a major contributor to elevated biomarker profile. Zambian preinfection individuals with positive Schistosomiasis antibody titers do not have significantly higher cytokine levels than individuals with negative Schistosomiasis antibody titers regardless of uninfected or preinfection status. Levels of biomarkers were compared between three groups: uninfected individuals with negative antibody titers (U-, light blue), preinfection individuals with negative antibody titers (P-, pink), uninfected individuals with positive titers (U+, dark blue) and preinfection individuals with positive antibody titers (P+, magenta) (Kolmogorov-Smirnov test, two-tailed, unadjusted p-values). [file 12977_2021_552_MOESM8_ESM.pdf]

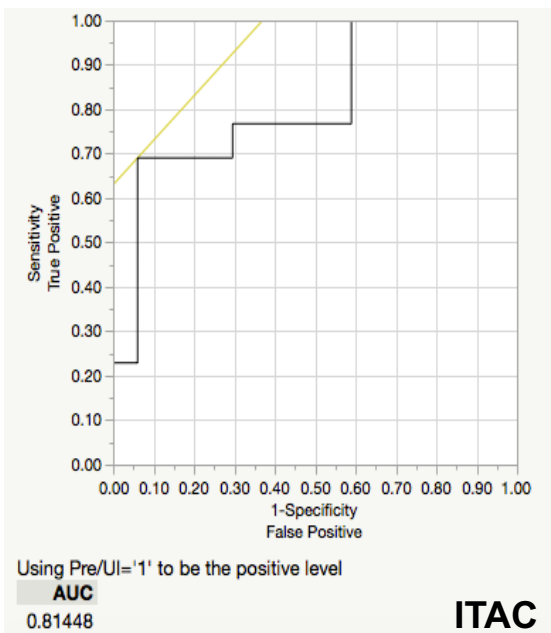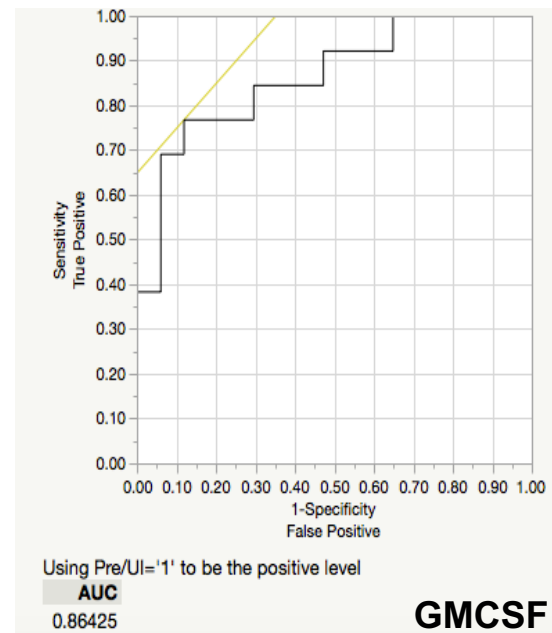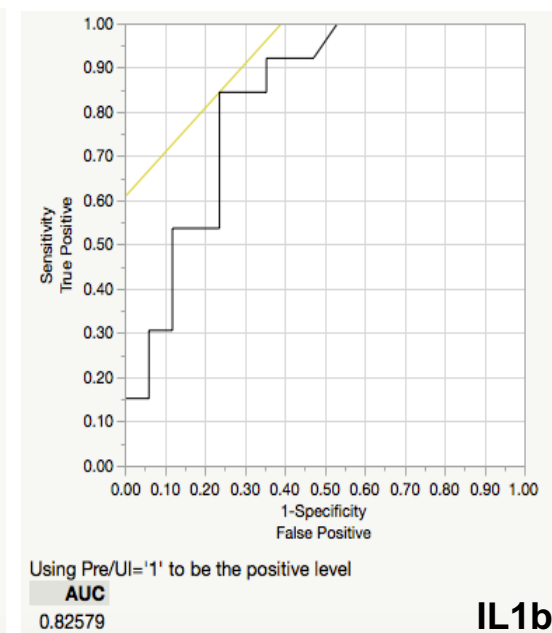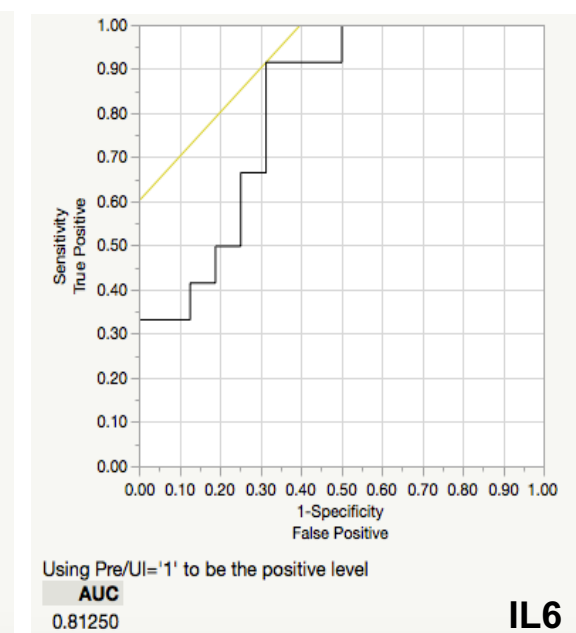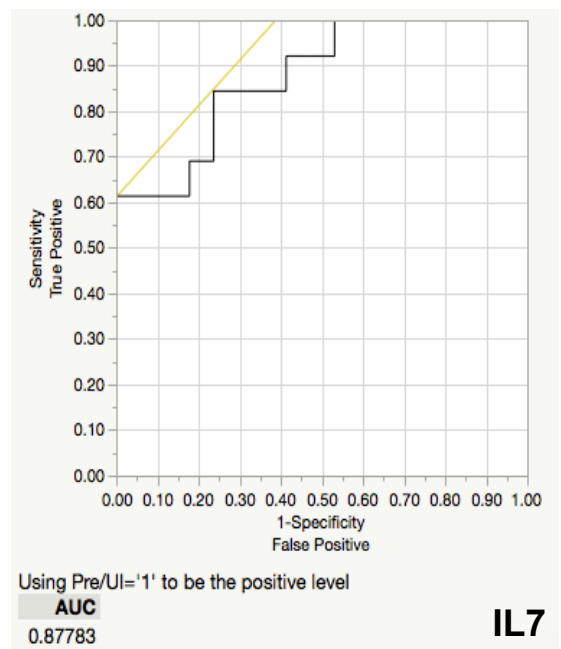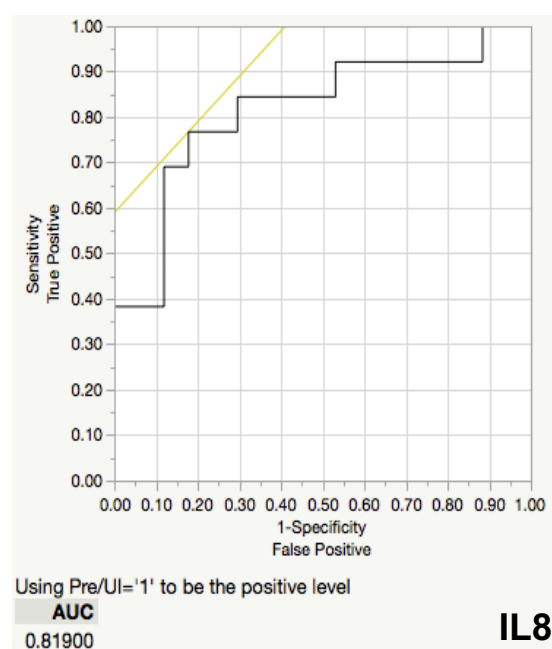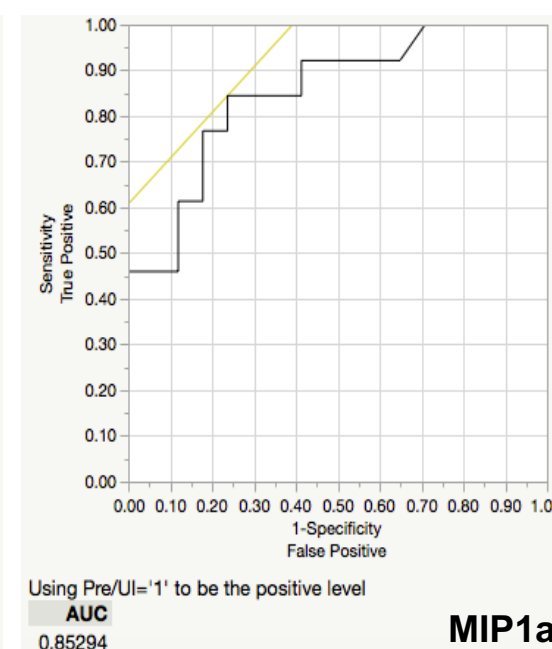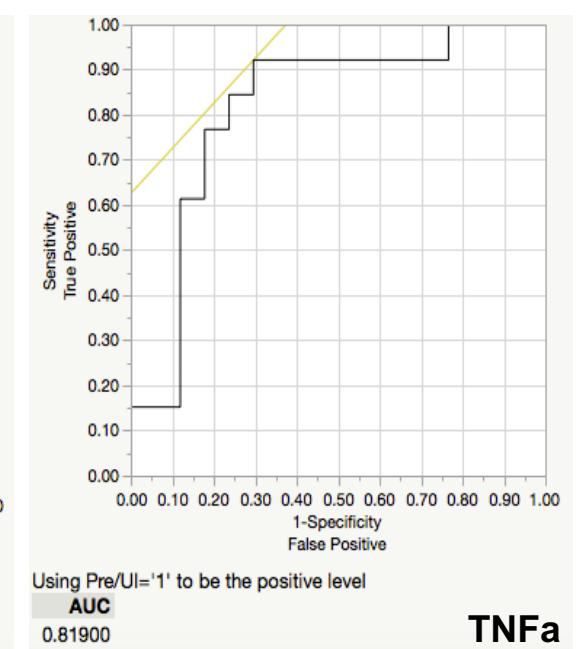

Supplement: Supplementary file 10 — Additional file 10: Figure S6. Receiver Operating Characteristics (ROC) curves for Rwanda cohort identifies biomarkers that distinguishes preinfection individuals. Elevated levels of ITAC, GMCSF, IL-1b, IL-6, IL-7, IL-8, MIP-1a, and TNFa identify individuals as risk for HIV acquisition. Area under the curve (AUC) shut off was 0.8 for separating the uninfected and preinfection individuals. [file 12977_2021_552_MOESM10_ESM.pdf]
