# Supplementary material for: Elevated levels of inflammatory plasma biomarkers are associated with risk of HIV infection
Source: Retrovirology. 2021 Mar 17;18:8. doi: 10.1186/s12977-021-00552-6 (PMC7968240; doi:10.1186/s12977-021-00552-6)
Supplement: Supplementary file 3 — Additional file 3: Table S2. Biomarkers increased in preinfection group compared to uninfected group in combined Rwandan and Zambian cohort. [file 12977_2021_552_MOESM3_ESM.pdf]

	Uninfected		Preinfection		
	Median (pg/ml)	IQR (pg/ml)	Median (pg/ml)	IQR (pg/ml)	FDR p-value
Fractalkine	71.42	23.67	99.15	38.73	0.00042
GM-CSF	10.8	6.255	18.76	9.9425	0.0021
ITAC	13.17	7.985	38.67	33.7325	0.00042
IL-1b	0.42	0.205	0.67	0.4575	0.00105
IL-2	1.3	0.56	2.03	1.6275	0.00289
IL-5	3.12	1.81	4.23	2.21	0.01431
IL-6	1.13	1.72	2.37	1.565	0.00382
IL-7	5.01	1.775	8.385	3.3525	0.00042
IL-8	2.52	2.355	5.465	3.33	0.00042
IL-10	12.41	9.2	19.365	13.2275	0.00574
IL-12	1.415	0.8725	1.88	1.1425	0.02532
IL-17a	4.75	5.895	7.475	3.695	0.00378
IL-21	1.43	1.33	2.4	1.5225	0.02112
IL-23	82.23	56.245	134.775	88.9275	0.00465
MIP-1a	18.17	6.965	23.685	7.885	0.00373
MIP-1b	3.85	2.06	6.18	7.885	0.00465
MIP-3a	7.11	5.065	9.54	4.78	0.00465
TNFa	1.8	1.005	3.015	1.985	0.00042
IFNg	7.21	5.24	8.56	5.0825	0.1155
IL-4	15.3	7.22	18.495	16.03	0.15561
IL-13	2.56	2.53	2.705	2.14	0.8026
