# Supplementary material for: Elevated levels of inflammatory plasma biomarkers are associated with risk of HIV infection
Source: Retrovirology. 2021 Mar 17;18:8. doi: 10.1186/s12977-021-00552-6 (PMC7968240; doi:10.1186/s12977-021-00552-6)
Supplement: Supplementary file 4 — Additional file 4: Table S3. Variable Importance values for Partial Least Square analyses for combined cohorts (A), Rwandan cohort (B), and Zambian cohort (C). [file 12977_2021_552_MOESM4_ESM.pdf]

(A)<br>Biomarkers	VIP	(B)<br>Biomarkers	VIP	(C)<br>Biomarkers	VIP
Fractalkine	1.3259	Fractalkine	0.9901	Fractalkine	0.2223
GM-CSF	1.0385	GM-CSF	1.301	GM-CSF	0.4878
IFNy	0.8451	IFNy	0.9651	IFNy	0.7346
IL-1b	1.1681	IL-1b	1.0939	IL-1b	0.4392
IL-2	1.0777	IL-2	1.2672	IL-2	0.4389
IL-4	0.3220	IL-4	0.1382	IL-4	0.0978
IL-5	1.0302	IL-5	0.8081	IL-5	1.5860
IL-6	0.7652	IL-6	0.9391	IL-6	0.6227
IL-7	1.3083	IL-7	1.5326	IL-7	0.9155
IL-8	1.1564	IL-8	1.2471	IL-8	2.0761
IL-10	0.9766	IL-10	0.8721	IL-10	0.4507
IL-12 (p70)	0.8602	IL-12 (p70)	0.7555	IL-12 (p70)	0.6799
IL-13	0.5169	IL-13	0.453	IL-13	0.3421
IL-17a	0.9232	IL-17a	0.8391	IL-17a	0.6320
IL-21	0.8949	IL-21	0.9531	IL-21	0.5320
IL-23	0.9879	IL-23	0.6269	IL-23	0.6380
ITAC	1.5373	ITAC	1.3263	ITAC	2.7488
MIP-1a	0.5792	MIP-1a	1.2595	MIP-1a	1.1830
MIP-1b	1.1439	MIP-1b	1.0304	MIP-1b	0.7871
MIP-3a	0.3005	MIP-3a	0.0568	MIP-3a	0.0651
TNFa	1.1986	TNFa	1.0876	TNFa	0.5248
