# Supplementary material for: Elevated levels of inflammatory plasma biomarkers are associated with risk of HIV infection
Source: Retrovirology. 2021 Mar 17;18:8. doi: 10.1186/s12977-021-00552-6 (PMC7968240; doi:10.1186/s12977-021-00552-6)
Supplement: Supplementary file 5 — Additional file 5: Table S4. Biomarkers increased in preinfection group compared to uninfected group in Rwandan cohort (A) and the Zambian cohort (B). [file 12977_2021_552_MOESM5_ESM.pdf]

A	Uninfected Median (pg/ml)	IQR (pg/ml)	Preinfection Median (pg/ml)	IQR (pg/ml)	FDR p-value
Fractalkine	77.48	17.79	92.39	29.16	0.0387
GMCSF	9.37	3.06	15.12	8.08	0.0287
IL-1β	0.38	0.16	0.62	0.26	0.0287
IL-6	0.99	1.4725	2.47	1.295	0.0352
IL-7	5.12	1.46	8.09	4.18	0.0287
IL-8	2.54	1.29	5.07	3.95	0.0339
ITAC	13.87	8.42	31.21	21.18	0.0287
MIP-1α	15.04	4.06	22.54	8.87	0.0287
TNF-α	1.74	0.98	2.95	0.92	0.0287

B	Uninfected Median (pg/ml)	IQR (pg/ml)	Preinfection Median (pg/ml)	IQR (pg/ml)	FDR P-value
Fractalkine	64.67	15.685	106.78	45.15	0.0021
IL-7	5.01	2.165	8.47	2.955	0.0042
IL-8	2.5	2.9625	5.82	2.395	0.0026
IL-23	79.03	45.565	146.59	65.405	0.0021
ITAC	12.3	5.8075	45.81	59.255	0.0021
TNF-α	1.86	0.9025	3.12	2.045	0.0119
