# Supplementary material for: Elevated levels of inflammatory plasma biomarkers are associated with risk of HIV infection
Source: Retrovirology. 2021 Mar 17;18:8. doi: 10.1186/s12977-021-00552-6 (PMC7968240; doi:10.1186/s12977-021-00552-6)
Supplement: Supplementary file 9 — Additional file 9: Figure S5. Receiver Operating Characteristics (ROC) curves for Zambia cohort identifies biomarkers that distinguishes preinfection individuals. Elevated levels of ITAC, Fractalkine, IL-23, IL-7, IL-8, and TNFa identify individuals as risk for HIV acquisition. Area under the curve (AUC) shut off was 0.8 for separating the uninfected and preinfection individuals. [file 12977_2021_552_MOESM9_ESM.pdf]

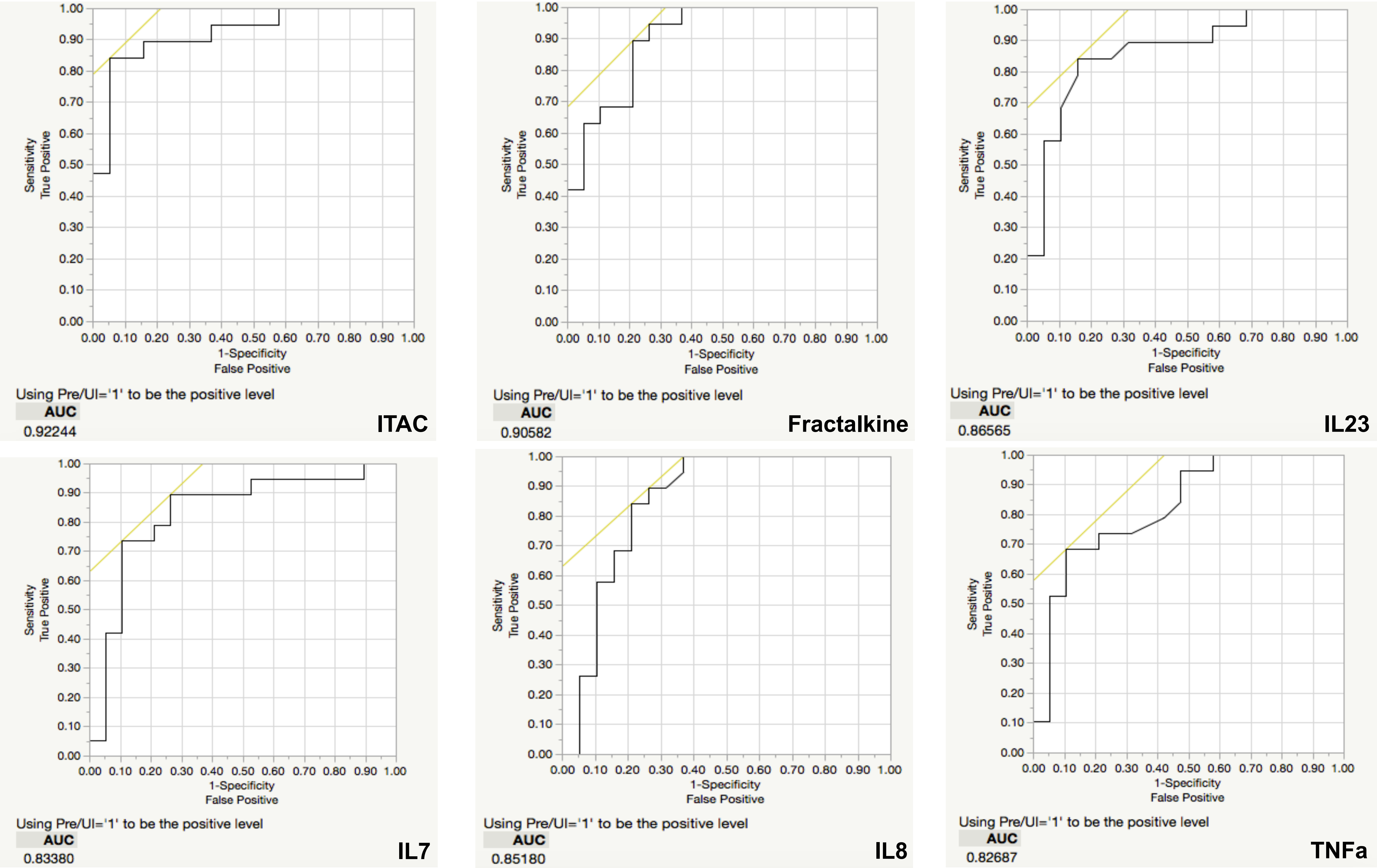
Sensitivity
True Positive
1-Specificity
False Positive
Using Pre/UI='1' to be the positive level
AUC
0.92244
ITAC
Using Pre/UI='1' to be the positive level
AUC
0.90582
Fractalkine
Using Pre/UI='1' to be the positive level
AUC
0.86565
IL23
Using Pre/UI='1' to be the positive level
AUC
0.83380
IL7
Using Pre/UI='1' to be the positive level
AUC
0.85180
IL8
Using Pre/UI='1' to be the positive level
AUC
0.82687
TNFa
