# Supplementary material for: Elevated levels of inflammatory plasma biomarkers are associated with risk of HIV infection
Source: Retrovirology. 2021 Mar 17;18:8. doi: 10.1186/s12977-021-00552-6 (PMC7968240; doi:10.1186/s12977-021-00552-6)
Supplement: Supplementary file 11 — Additional file 11: Table S5. Elevated biomarkers and diseases/infections mentioned in Discussion. [file 12977_2021_552_MOESM11_ESM.pdf]

Disease or Infection	Elevated biomarkers
**Hepatitis C Virus and HIV**	IL-1b, IL-6, TNFa, IL-8, CXCL10 (22)
**Hepatitis C Virus**	IFNg, IL-17 (21, 22)
**P. falciparum**	TNFa, IFNg, IL-1, IL-6, IL-12, IL-4, sTNFRI, sTNFRII (25, 26)
**P.vivax**	IL-6, IL-17, IL-12, TNFa, MCP1, CRP, IFNg (27, 28)
**Tuberculosis**	IL-10, IL-1a, IP-10, MCP1, TNFa, IL-2, IFNg, GMCSF, IL-3, IL-13, MIP-1b, IL-5, IL-10, VEGF (29-32)
**S. stercoralis and tuberculosis**	IL-5, IL-13, IL-17, IL-22, IL-10 (34)
**Type 2 Diabetes**	Fractalkine (41)
**Systemic Lupus Erythematous**	Fractalkine (40)
**Systemic Sclerosis**	Fractalkine, ITAC, CXCL10 (42)
**Inflammatory Bowel Disease**	ITAC (43)
**Sarcoidosis**	ITAC (43)
**Fibromyalgia**	ITAC, IL-7 (46)
**Colorectal and Esophageal Cancer**	IL-7 (47)
